# A Comprehensive Review of Artificial Intelligence and Colon Capsule Endoscopy: Opportunities and Challenges

**DOI:** 10.3390/diagnostics14182072

**Published:** 2024-09-19

**Authors:** Joana Mota, Maria João Almeida, Francisco Mendes, Miguel Martins, Tiago Ribeiro, João Afonso, Pedro Cardoso, Helder Cardoso, Patricia Andrade, João Ferreira, Guilherme Macedo, Miguel Mascarenhas

**Affiliations:** 1 Precision Medicine Unit, Department of Gastroenterology, São João University Hospital, 4200-427 Porto, Portugal; 2 WGO Gastroenterology and Hepatology Training Center, 4200-427 Porto, Portugal; 3 Faculty of Medicine, University of Porto, 4200-427 Porto, Portugal; 4 Department of Mechanical Engineering, Faculty of Engineering, University of Porto, 4200-465 Porto, Portugal; 5 Digestive Artificial Intelligence Development, 4200-135 Porto, Portugal; 6 ManopH Gastroenterology Clinic, 4000-432 Porto, Portugal

**Keywords:** colon capsule endoscopy, artificial intelligence, convolutional neural network

## Abstract

Colon capsule endoscopy (CCE) enables a comprehensive, non-invasive, and painless evaluation of the colon, although it still has limited indications. The lengthy reading times hinder its wider implementation, a drawback that could potentially be overcome through the integration of artificial intelligence (AI) models. Studies employing AI, particularly convolutional neural networks (CNNs), demonstrate great promise in using CCE as a viable option for detecting certain diseases and alterations in the colon, compared to other methods like colonoscopy. Additionally, employing AI models in CCE could pave the way for a minimally invasive panenteric or even panendoscopic solution. This review aims to provide a comprehensive summary of the current state-of-the-art of AI in CCE while also addressing the challenges, both technical and ethical, associated with broadening indications for AI-powered CCE. Additionally, it also gives a brief reflection of the potential environmental advantages of using this method compared to alternative ones.

## 1. Introduction

Conventional colonoscopy (CC) remains the most commonly utilized exam to evaluate colonic diseases, both for diagnostic and therapeutic purposes [1]. Indeed, the number of colonoscopies is increasing globally, and the specificities of this exam keep being analyzed [2]. In the present day, we find ourselves in a world where patients are demanding high-quality and convenient healthcare services (faster and painless exams, fewer hospital visits, etc.), while seeking more economical and ecologically beneficial solutions to solve their problems. This reflects their desire to take more control over the management of their own health [3]. 

The advent of capsule endoscopy (CE) for the study of small bowel (SB) diseases and its successful results brought to light the possibility of evaluating other anatomic locations, the colon not being an exception, in a more convenient and patient-friendly manner, potentially overcoming the drawbacks of CC. 

**Figure 1:**
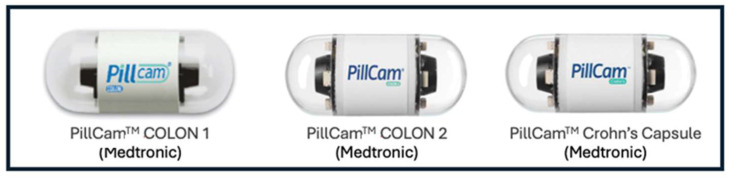
Examples of colon capsule endoscopy devices.

The first generation of colon capsule endoscopy (CCE), CCE-1 (Pill Cam Colon, Given Imaging, Yokneam, Israel), was introduced in 2006 by Eliakim et al. [4]. Since then, there have been alterations and improvements, both in hardware and software, that aim to enhance its performance. In 2009, a second-generation CCE was developed, CCE-2 (PillCam Colon 2, Given Imaging, Yokneam, Israel). More recently, in 2017, a later-generation panendoscopy device, named PillCam Crohn’s Capsule (Medtronic, Dublin, Ireland), as it was designed for the assessment of the entire length of the gastrointestinal (GI) disease in Crohn’s disease (CD) patients, was released [5,6]. Figure 1 illustrates the aforementioned colon capsule endoscopy devices. Later, other types of CCE became available [7]. 

Currently, the spectrum of indications for CCE is very narrow, and, to date, it is only validated for patients who cannot undergo CC or in cases when it is not feasible to perform the exam. For instance, the role of CCE in the diagnosis and follow-up of suspected or confirmed IBD is still not established [8,9]. Nevertheless, CCE has only a few contraindications and is highly accepted by patients due to its non-invasive, painless, and sedation-free nature. Despite the possibility of adverse effects, namely capsule retention, these are rare and can be easily minimized by previously performing a patent capsule exam [10].

However, by displaying two cameras, each examination generates a high number of frames, adding to the task complexity and extending the time required for video analysis, with reading times reaching up to 120 min, further increasing the overall workload of physicians [11]. Indeed, CCE video reading is a laborious task that is prone to error, requiring specific training to minimize this negative impact on its diagnostic accuracy [5]. Additionally, there is a need to establish the optimal bowel cleansing preparation for improving colon mucosa visualization and the rate of complete exams [12].

Artificial intelligence (AI) application in the healthcare industry is witnessing exponential growth [13]. CCE stands as one of the fields that can greatly benefit from applying AI tools for video reading assistance. Indeed, by enabling a more cost-effective examination while upholding a high level of accuracy, AI-powered CCE can pave the way for expanding the range of clinical indications of this diagnostic modality [9].

This review provides an overview of the current reported applications of AI in the scope of CCE and briefly summarizes some technical and ethical limitations that still hinder its wider adoption.

## 2. The State of the Art in Colon Capsule Endoscopy

### 2.1. AI and Protruding Lesions

Colorectal cancer (CRC) remains a huge concern within the medical community due to its substantial burden [14,15]. CRC is one of the most preventable forms of cancer if colorectal polyps are detected and removed early in the process [14,16]. Due to their clinical significance, the identification of polyps is one of the areas that has garnered the most attention from researchers.

There are a multitude of screening options available, and the screening programs differ globally [16]. The impact of CC is well recognized in the natural history of colorectal cancer. Indeed, a recent randomized trial concluded that the risk of colorectal cancer within ten years is lower among those who undergo screening with CC [17].

However, CC has some downsides that limit its use as a populational screening method, namely, the highly invasive nature of the procedure, the lack of patient friendliness, and the inherent and well-known risks of the procedure. While serious adverse effects are uncommon during surveillance colonoscopies, minor/transient symptoms are much more common [18]. The need to develop other equally effective screening methods to mitigate the great demand for CC has opened the path for CCE as a safe and effective tool.

Another major limitation hindering its use is the extensive preparation necessary for a comprehensive surveillance CCE exam. Indeed, CCE requires a more extensive bowel preparation compared to CC, which discourages its use, despite having good accuracy; this topic will be further explored ahead [19].

Like its impact in other domains, AI could alleviate some major limitations while achieving highly satisfactory results.

The detection of polyps poses a significant challenge due to various factors. The drawbacks include the presence of debris, liquids, or bubbles; the difficulty in distinguishing between the geometry of polyps and the folds of healthy intestinal mucosa; and, perhaps the most crucial factor, the lack of consistent morphology, size, texture, and color features among polyps, even within the same patient [20]. The variable lighting and its infrequent presence in a given CCE video make it challenging to develop a robust method for reliable polyp detection [21].

Blanes-Vidal and colleagues were the pioneers in assessing the performance of convolutional neural networks (CNNs) for the autonomous detection of polyps in CCE images (PillCam Colon 2). The researchers gathered images of polyps identified by CCE (*n* = 375) from 255 patients that had a positive fecal immunochemical test (FIT) in a national screening program in Denmark and compared them with results from CC. They integrated these images into a modified version of the existing CNN, AlexNet, which exhibited remarkable accuracy (96%), sensitivity (97%), and specificity (93%) in detecting colorectal polyps [22].

The latter study gave rise to a subsequent sub-study in which an improved version of a ZF-Net CNN was created to localize regions within images containing colorectal polyps. The developed CNN had accuracy of 98.0%, sensitivity of 98.1%, and specificity of 96.3%, outperforming the available evidence. Additionally, the study provided greater interpretability to the network’s predictions by studying saliency maps where the contribution of polyps in terms of activation was significant [14].

In a similar approach, Gilabert, P. et al. developed a novel CNN-based system, AI-tool, to assist in colonic polyp detection in CCE videos and compared it to the classical linear review method (RAPID Reader Software v9.0). For each of the eighteen videos, the system generated a probability score per image frame to contain a polyp and a heatmap explaining the reasoning for the prediction [21]. Similar to saliency maps mentioned before, this heat map allowed experts reviewing the video to focus on the specific area of the image where the CNN suggested the polyp was located, thus reducing the cognitive load of the reviewer. By implementing this new strategy, the reviewing time was reduced by a factor of six, and the sensitivity to polyp detection increased from 81% to 88%. Furthermore, the results indicated that their system was also particularly effective in detecting small polyps or polyps that appear only briefly in a few frames, thereby enhancing the clinical viability of CCE as an alternative method for examining the GI tract [21].

A Portuguese group developed a CNN-based algorithm for automatically detecting colonic protruding lesions (including polyps, epithelial tumors, and subepithelial lesions) in the colonic lumen using CCE images. Out of 124 enrolled patients, 5715 frames were extracted. Among them, 2410 displayed protruding lesions, and 3305 depicted normal mucosa and other findings. In general, the developed model exhibited high levels of sensitivity (90%), specificity (99.1%), and overall accuracy (95.3%) in detecting protruding lesions, accomplishing it with a remarkable image processing performance, and being capable of reading sixty-five images per second [23]. The same group developed a CNN that detected protruding lesions with sensitivity and specificity of 90.7% and 92.6%, respectively [24].

Many more deep learning (DL) methods to deal with polyp recognition challenges are described in the literature, mainly based on CNNs, each attaining high performance in classification tasks [25,26,27,28,29,30]. However, providing a detailed description of each method is beyond the scope of this article.

Some studies also place attention on detecting stages of colorectal neoplasia. An example is a study in which the authors developed an AI model for the automatic detection of colorectal neoplasia in CCE colon images. Using data from 178 patients with colorectal neoplasia and 6 normal patients, 20,717 frames were divided for training and validation datasets. This study reported sensitivity, specificity, and an area under curve (AUC) of 79%, 87%, and 0.90, respectively, for detecting colorectal polyps and cancers [31].

### 2.2. AI and Inflammatory Bowel Activity

The CD is a panenteric disease and likewise requires a tool capable of investigating the entire GI tract. Assessing the extent and severity of the inflammation is crucial in guiding therapy, particularly when considering medication discontinuation. Therefore, a single-time panenteric evaluation (preventing the need for invasive endoscopic exams) is appealing in the context of IBD management, as the concept of treat-to-target often requires frequent and seriated endoscopic assessments to evaluate mucosal healing in response to different treatment strategies [32]. Since its introduction, CCE has allowed the direct, detailed, and simultaneous evaluation of both enteric and colonic mucosa, with a higher number of lesions detected when compared to standard CE [33].

Due to better tolerance compared to CC, CCE increases patient compliance and enables adequate surveillance in these populations. Nonetheless, the role of panenteric CE is yet to be established, even though it is already acknowledged that it could play a major part in future algorithms for the non-invasive diagnosis and monitoring of CD.

The implementation of AI algorithms for the automatic assessment of inflammatory burden (mainly by the detection of erosions and ulcers) in CE has achieved promising results. However, the impact of these technologies in pan-endoscopy is still being determined.

In 2021, a Danish group examined for the first time the use of DL for the detection and classification of the severity of lesions associated with CD (normal mucosa, nonulcerated inflammation, aphthous ulceration, ulcers, and extensive ulceration) in both the SB and colon through the application of an image preprocessing step with a texture enhancement method for capsule endoscopy images. In 7744 images collected (2772 from the colon) from 38 patients with clinically suspected or known CD, their automated framework diagnosed ulcerations with almost perfect sensitivity, specificity, and diagnostic accuracy (96%, 100%, and 98%, respectively) compared to a manual analysis by experts and with similar high diagnostic accuracy for the detection of ulcerations in the SB and colon (diagnostic accuracy was 99% for the SB and 98% for the colon). Moreover, the lesion characterization also showed substantial to almost perfect agreement with experts’ opinions (κ = 0.72), once again encouraging the future role of DL algorithms for the autonomous assessment of disease severity on CD [34].

Ferreira et al. presented a CNN framework for the automatic detection of both SB and colonic ulcers and erosions using Pillcam Crohn’s images. From 59 Pillcam Crohn’s exams from two centers in Portugal, a total of 24,675 frames of enteric or colonic mucosa were extracted, of which, 5300 displayed ulcers and erosions. The total pool was then split into training and validation datasets. Overall, the CNN had sensitivity, specificity, and accuracy of 98%, 99%, and 99%, respectively. The AUC for the detection of ulcers and erosions was approximately 1.00 [35].

The same group recently developed a pioneer CNN for automatically detecting and validating colonic ulcers and erosions in CCE images. Ultimately, 124 patients from two institutions were enrolled, and 37,319 colonic images were retrospectively extracted and reviewed from full-length CCE videos. The model’s performance in detecting these lesions was remarkably high, reaching sensitivity, specificity, and global accuracy of 97%, 100%, and 100%, respectively. The AUC for the distinction between colonic ulcers and erosions and normal mucosa was 1.00. Moreover, the CNN demonstrated high imaging processing performance. Considering the average rate of ninety frames per second, it was estimated that only nine minutes would be required for a full-length CCE video revision [36].

### 2.3. AI and Gastrointestinal Bleeding

The role of conventional CE in obscure GI bleeding is well established. Currently, panendoscopy is slowly starting to prove its value in this context. Emerging evidence suggests that the use of CCE as the initial endoscopic approach not only holds the potential to decrease the need for unnecessary colonoscopies in patients with melena and negative esophagogastroduodenoscopy but also aims to identify patients who would benefit from subsequent therapeutic endoscopy [37]. More recent evidence suggests the potential use of CCE even before esophagogastroduodenoscopy, and CC in high-risk patients may be helpful by selecting those who need therapeutic endoscopy, once again avoiding up to 70% of diagnostic endoscopies [38]. However, further research is required before making practical recommendations on these grounds.

**Figure 2:**
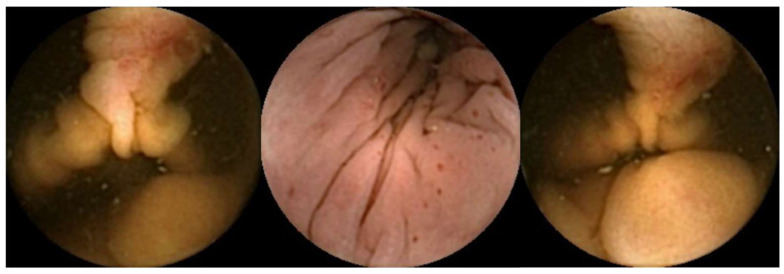
Vascular lesions automatically detected by AI models.

The use of AI for detecting blood content and a further source of bleeding in CCE images is in its early stages. To the best of our knowledge, there are only two studies on automatically detecting colonic luminal blood or hematic vestiges in CCE images. Figure 2 illustrates hematic lesions identified by AI models.

Mascarenhas et al. developed a pilot CNN for the automatic detection of blood or hematic residues in CCE images. From 24 patients, a total of 5825 frames were extracted, of which, 2975 contained blood. Their algorithm detected blood and hematic traces with high sensitivity, specificity, and accuracy (99.8%, 93.2%, and 96.6%, respectively) [39].

The latter study was followed by a multicentric study in which the authors designed a trinary network aiming to detect and differentiate normal colonic mucosa, blood, and hematic residues as well as mucosal lesions (including ulcers, erosions, vascular lesions (red spots, angioectasia, and varices), and protruding lesions (polyps, epithelial tumors, submucosal tumors, and nodes)). For its development, a total of 9005 images were collected from 124 patients. Globally, the CNN had sensitivity and specificity of 96% and 98%, respectively, providing accurate predictions at 98%. Additionally, with sensitivities and specificities of over 90%, the network could detect and differentiate each category effectively. This article highlights the main advantage of using AI in panendoscopy: enabling the simultaneous detection of different lesions in a single analysis of the entire GI tract in a highly sensitive and effective manner [40].

By improving its diagnostic yield and time efficiency, the use of AI in CCE images can pave the way for potential recommendations in investigating lower GI bleeding, particularly when CC is contraindicated or not desired by the patient.

### 2.4. Improving Bowel Preparation for Colon Capsule Endoscopy

The significance of colon cleansing plays a crucial role regarding the implementation of CCE as an alternative diagnostic method to CC. This significance is related to two components that are currently not entirely addressed: a scoring system for assessing cleansing quality and an optimal preparation protocol to achieve adequate bowel preparation.

On the one hand, there is a need for a reliable, objective, and reproducible method for bowel cleansing quality assessment, capable of certifying a CCE as conclusive and ensuring a minimum quality that allows physicians to have confidence in the detected anomalies, and perhaps even more crucially, in their absence [5]. Moreover, unacceptable bowel cleansing in the CCE exam warrants the need for a diagnostic CC [41]. Therefore, an adequate cleansing quality avoids the need of having to revert to diagnostic CC, which is convenient for patients (need for new bowel preparation, delayed diagnosis), caretakers (limited resources, waiting lists, higher costs), and the environment (reducing the number of exams needed).

**Figure 3:**
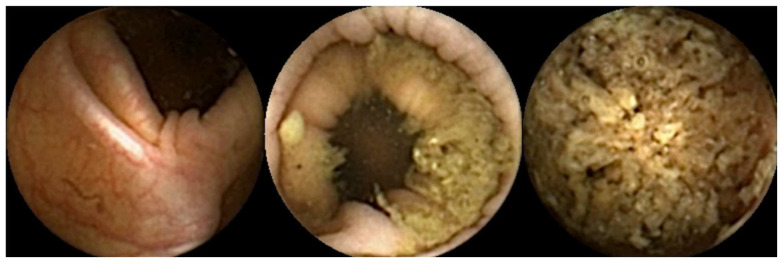
Images illustrating different qualities of colon capsule endoscopy preparation.

Furthermore, there is currently no scoring system that meets the requirements of being time-efficient, consistent, and, most importantly, devoid of inter-observer variability to assess colon cleanliness. Figure 3 illustrates different qualities of preparation in CCE.

A recent study found inter- and intra-observer agreement to be lower on assessing bowel cleansing quality than on polyps’ detection and that professional experience did not seem to bring any benefit in that regard, further reinforcing the high heterogeneity in how experts analyze mucosa cleaning [42].

The Leighton–Rex scale is the sole published method for assessing it in CCE, categorizing five bowel segments into four distinct qualitative cleansing levels [43]. Nevertheless, it lacks validation and heavily relies on the reader’s clinical judgment. Colon Capsule Cleansing Assessment and Report (CC-CLEAR) is a novel scale that instead divides the colon into three segments and performs a quantitative assessment of the percentage of visualized mucosa, presenting a far superior agreement rate to the previously described Leighton–Rex scale [44].

Integrating automated bowel cleansing classification into AI algorithms aiding in the reading of CE examinations would be the next critical step for its smooth application in clinical practice. To achieve this, a reliable input, on which the model will be trained, must be provided.

Recently, a DL model was developed to distinguish different levels of colon preparation based on a simple three-level classification scale of cleanliness defined through the relative proportion of the mucosa that was visualized in each image, reporting accuracy of 95%, sensitivity of 91.4%, and specificity of 96.8% [45].

On the other hand, an optimal bowel preparation protocol that maximizes the diagnostic performance of CCE has also been a matter of debate. Research has consistently demonstrated that enhancing bowel cleaning increases the sensitivity and specificity of CCE as a diagnostic option, namely for colorectal polyp detection [46]. However, the inherent technical inability to perform intraprocedural actions such as washing or aspirating colon debris during CE, unlike CC, renders the technique highly dependent on the quality of bowel preparation. Numerous studies, including systematic reviews and meta-analyses, have been conducted to establish the optimal bowel preparation protocol, but a definitive conclusion remains elusive [12,47,48,49,50]. This is primarily attributed to the considerable heterogeneity among studies, which is an expectation given the varied criteria used to assess the quality of bowel preparation (reinforcing the earlier point even further), differences in administration timing for various components in protocols, and the stepwise nature of distinct bowel cleansing protocols. The most recent evidence indicates that the most favorable rates of adequate bowel cleansing were achieved when employing a low-fiber diet on the day preceding the CCE exam, coupled with premedication using an adjunctive laxative like sennosides, the routine administration of a prokinetic agent before capsule ingestion, a split dose of <4 L PEG as the purgative solution, and a gastrografin-based booster [47]. However, patient tolerability, a key factor to evaluate the adherence and applicability of a preparation regime in clinical practice, is only concluded speculatively in most studies, given their retrospective design.

Taking this into account, it is necessary to develop large clinical trials on different bowel preparations, as a lot of work remains to be done in this area in order to level CCE as an equivalent to CC.

## 3. Environmental and Ecological Impact of AI-Powered Colon Capsule Endoscopy

The healthcare sector is globally responsible for a great amount of greenhouse gas emissions, even though its environmental impact has been overlooked. The influence of this sector on global warming will increase as the need for care rises [51]. The civilizational awareness is becoming increasingly sensitive to issues of ecological and environmental natures, and embracing greener practices seems to be an evolving concern in every aspect of today’s society, with healthcare being no exception [52,53,54]. Efforts to reduce carbon footprints can have major economic and health benefits [51].

**Figure 4:**
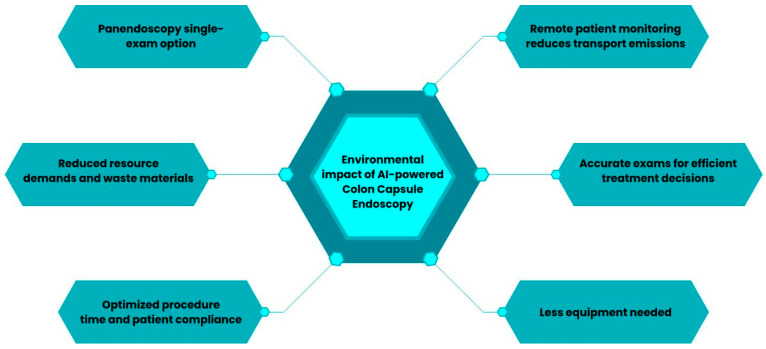
The positive environmental impact of using artificial intelligence in colon capsule endoscopy.

This happens to be a particularly pressing issue in the field of gastroenterology. AI-powered CCE appears to be a greener method for evaluating the GI tract and therefore a greater contributor to reducing the ecological footprint of the specialty. Some advantages of AI-powered CCE are illustrated in Figure 4.

Firstly, as described above, the higher accuracy of the methods will allow for a reduction in the number of exams performed and fewer repetitions of such examinations [53]. It may also decrease the need for histopathology evaluation, but further studies are still needed. Remote monitoring has the potential to not only reduce healthcare expenses but also reduce transport-related footprints. Indeed, the reduction in transport emissions and air pollution stands as one of the primary goals of the United Nations Environmental Programme.

**Figure 5:**
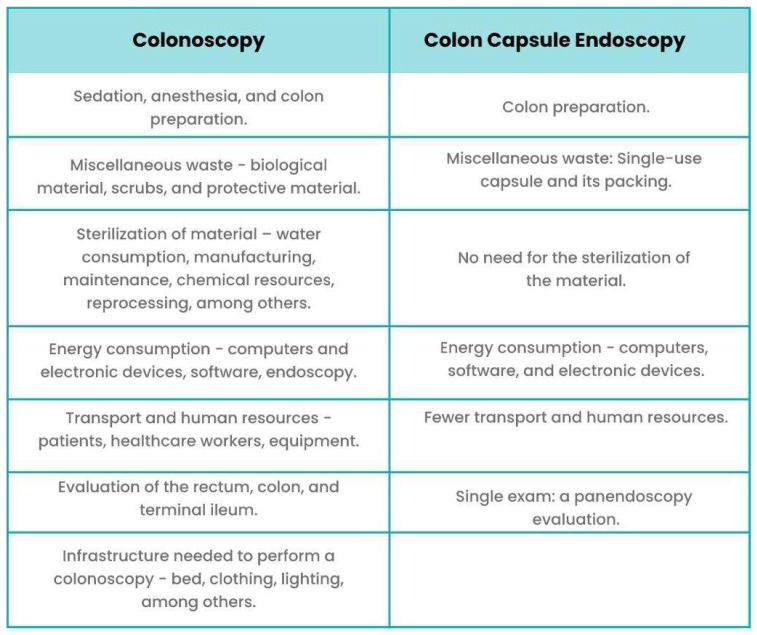
Major environmental and ecological differences between colonoscopy and colon capsule endoscopy.

Additionally, it also allows for reduced resource demands and waste materials. CCE, being a biodegradable device with a length of approximately 30 mm, naturally generates less waste compared to other techniques. Furthermore, being a non-invasive procedure, it does not require sedation or other additional human and material resources (such as a recovery room after CC) [53,55]. Figure 5 highlights the major differences between CC and CCE.

Another promising approach involves the concept of a minimally invasive panendoscopy, offering a single examination option to assess the entire GI tract. Therefore, decreasing the need for multiple procedures (endoscopy, colonoscopy, and enteroscopy) seems to be an important target for reducing the environmental impact [53].

## 4. Ethical Challenges in AI-Powered Colon Capsule Endoscopy

Prior to the widespread acceptance of AI, it is essential to address some bioethical challenges. These challenges span three decisive moments in AI implementation in clinical practice: at the data-acquisition level (input), during model development (AI tool itself), and at the AI-generated response level (output) [56].

Healthcare data are the driving force behind each AI deployment in medicine. With the rising prevalence of medical AI applications and the broader collection and exchange of data, in addition to growing cybersecurity challenges, privacy concerns arise [54,57,58]. Therefore, solutions ensuring non-traceability and confidentiality (e.g., de-identification techniques), along with adherence to regulatory guidelines (e.g., General Data Protection Regulation 2016/79 in E.U), are evidently necessary [58,59]. Recent initiatives related to the use of blockchain technology may also mitigate these concerns in data handling and management, given its chronological and immutable approach to storing information [60,61].

Regarding the AI model itself, we must deal with the risk of introducing certain biases, either through the training data or during the algorithm’s design process, that could affect the transferability of our model to different data. One particular bias that deserves attention is selection bias [62]. AI systems are tailored-made, meaning that they are shaped by the data on which they are trained and validated [57]. Therefore, when these training databases are not representative of the target population for whom the test is intended, we may introduce selection bias that impacts its external validity. Additionally, AI models are also susceptible to overfitting, a phenomenon that occurs when the model is so closely tuned to the training data that it does not yield equivalent diagnostic performance when exposed to different data [63].

According to what the model’s output stands for, the main issues raised are those of explainability and accountability. When explainable, the model’s reasoning behind the attribution of a pathological meaning to a specific image is readily understood. In the absence of this explainability, decisions based on AI face greater resistance. Regarding accountability, in the event that a clinical decision leads to harm to the patient, it is still debatable whether a physician should be held accountable for a clinical decision made by an AI model if such a decision results in harm to the patient [56].

## 5. Concluding Remarks and Future Directions

The integration of AI into CCE seems to represent a breakthrough in medical diagnosis. The current evidence has demonstrated remarkable results on sensitivity, specificity, and AUC, showing great promise in the detection of colonic diseases with reduced reading time. The effectiveness and efficiency of AI algorithms are likely to expedite clinical diagnosis, prompting rapid medical responses and contributing to the standardization of medical care by minimizing variability among medical professionals and institutions. Therefore, addressing the ongoing challenges, such as ethical problems, seems to be the next logical step.

There is no doubt that the application of AI models in CCE will play a pivotal role in assisting physicians in their decision-making processes in the future. Prospective research articles and further technological developments are essential to place CCE powered by AI in a comparable position to the current gold standard on colonic investigations, conventional colonoscopy.

What we expect: AI-powered CCE is expected to attain higher diagnostic accuracy in a time-efficient way, ultimately spanning the indications for its use and leading to improved healthcare outcomes.

What we have: CCE is a non-invasive and patient-friendly diagnostic modality, yet its indications are sparse due to its lengthy reading times, its inherent inability to perform intra-procedure actions, and its need for more rigorous bowel cleansing protocols. Nevertheless, already-developed CNN models showed great promise in employing AI in CCE, with the majority achieving sensitivity and specificity rates higher than 90% and AUC close to one.

What is coming: In light of the promising results obtained from applying AI to assist in reading CCE exams, there is a general belief that the indications for performing CCE may increase (particularly as an equally valid option to colonoscopy for CRC screening), thanks to the enhanced diagnostic accuracy that AI models add. Looking ahead to the future, there is a long journey to maximize the efficacy of this technology, and some technological and ethical issues that persist should be addressed in the next decade.
